# Recognizing Physical Activity of Older People from Wearable Sensors and Inconsistent Data

**DOI:** 10.3390/s19040880

**Published:** 2019-02-20

**Authors:** Aimilia Papagiannaki, Evangelia I. Zacharaki, Gerasimos Kalouris, Spyridon Kalogiannis, Konstantinos Deltouzos, John Ellul, Vasileios Megalooikonomou

**Affiliations:** 1 Department of Computer Engineering and Informatics, University of Patras, 26504 Patras, Greece; papagianna@upatras.gr (A.P.); kalouris@ceid.upatras.gr (G.K.); kalogianni@ceid.upatras.gr (S.K.); deltouzos@upatras.gr (K.D.); vasilis@ceid.upatras.gr (V.M.); 2 Department of Neurology, University Hospital of Patras, 26504 Patras, Greece; ellul@upatras.gr

**Keywords:** activity recognition, support vector machine (SVM) classification, deep learning, convolutional neural networks, wearable devices, physiological monitoring

## Abstract

The physiological monitoring of older people using wearable sensors has shown great potential in improving their quality of life and preventing undesired events related to their health status. Nevertheless, creating robust predictive models from data collected unobtrusively in home environments can be challenging, especially for vulnerable ageing population. Under that premise, we propose an activity recognition scheme for older people exploiting feature extraction and machine learning, along with heuristic computational solutions to address the challenges due to inconsistent measurements in non-standardized environments. In addition, we compare the customized pipeline with deep learning architectures, such as convolutional neural networks, applied to raw sensor data without any pre- or post-processing adjustments. The results demonstrate that the generalizable deep architectures can compensate for inconsistencies during data acquisition providing a valuable alternative.

## 1. Introduction

The monitoring of daily activity patterns may support people seeking to enhance their personal fitness, promote patient engagement in the management of chronic diseases, or enable providers and patients to gain insights into the progression and impact of illnesses [1]. A great deal of research work has focused on the concept of frailty which has proven to affect older people’s lives and health radically [2], increasing the risk for falls, disability, hospitalization, loss of autonomy and mortality. Because of its complexity, multi-faceted nature and unclear pathophysiology, there is a great difficulty in defining, early identifying and preventing frailty. Information and communications technologies [3,4,5,6,7] try to address this unmet need, many of them by monitoring the physical behavior of older people aiming at providing solutions for active and healthy aging.

Most of the technological solutions previously reported in the literature for the recognition of activities of daily living (ADL) use a variety of wearable and non-wearable sensors. In this paper we present an activity classification scheme for detecting movement patterns of older people and focus on the necessary model reconfigurations for resolving challenges imposed by inconsistent measurements due to the type and misplacement of the sensors. Two approaches will be presented. The first is based on standard machine learning techniques coupled with necessary pre- and postprocessing steps to tackle data inconsistencies [8], which is referred to as the augmented standard approach in the paper. This approach was incorporated in the online analysis module of the FrailSafe project [9], and therefore was constrained to be computationally efficient. The second approach exploits deep network architectures, and specifically convolutional neural networks (CNNs), and aims to provide an alternative solution for unified multi-scale feature extraction and classification.

### 1.1. Related Work

One of the earliest and well cited studies on sensor-based ADL classification used accelerometers in multiple locations of the body of young individuals and performed decision tree classification [10]. The results showed that for some activities inter-subject analysis was difficult, requiring the training of subject-specific models. A signature extraction methodology was proposed in [11] using a smartphone’s accelerometer, placed at the subjects’ pelvis and implementing a threshold-based, or a principal component analysis (PCA)-based classification algorithm. In [12] the possibility of improving ADL classification accuracy by applying feature ranking and selection is explored. Activity recognition was performed in [13] using a hidden Markov model on recordings from sensors placed in the house and on the body, whereas in [14] the aim was to identify high falls’ risk-related activities of older people based on a wearable passive radio frequency identification sensor. Analysis was based on data from healthy adult volunteers.

Although many frameworks have been reported in the literature for activity monitoring of older people, most of them have been tested on data from young and healthy participants [11,12,14], or the experiments were performed in laboratory conditions, e.g., in [13] a scaled model of a house used along with a simulated sequence of activities. Those works report high classification accuracy, but results are not directly comparable with uncontrolled monitoring systems in real home environments. In contrast to the aforementioned studies, our approach is tested on data unobtrusively recorded from wearable sensors designed for monitoring the physiological signals of older people during their everyday life activities.

Comparable works that use wearable sensors on older people for activity recognition aim at providing the means for promoting active and healthy ageing through the development of assisted living systems or prediction of adverse events [15,16,17,18]. In [15] the authors reported the use of a smart watch enclosing three different kinds of sensors, namely accelerometer, temperature sensors and altimeter. After performing some calibrating actions on the raw signals and feature selection, neural network and support vector machine (SVM) classifiers were used for classifying the activities of the elderly. In [16] an inertial measurement unit located near the sternum and a thigh wearable sensor were used to detect the posture of the elderly with the deployment of a rule-based algorithm. Extraordinary work on activity classification for the elderly is reported in [17], where the idea of instrumented shoes able to record movement is introduced for the purpose of discriminating postural transitions, locomotion and walking activities using decision trees. A review on the potential benefits of ADL monitoring in the care of elderly people is presented by Gokalp and Clarke [18].

Deep learning architectures, such as convolutional neural networks (CNNs), have also been previously exploited for ADL recognition [19,20,21,22]. CNNs show great potential because they can tackle the two main elements of ADL recognition. The first refers to localization, i.e., the ability to capture only the part of the signal stream that is relevant to the pattern of interest. In fact, continuously monitored physiological signals contain in the majority non-specific or not distinct activities or transitional states. These irrelevant parts, denoted as null activity, dominate over the few distinct classes introducing a major challenge for classification. The second element is the rich variation in how a given activity can be performed, usually consisting of the decomposition into several movement patterns, arranged sequentially with a smooth transition. CNNs can extract discriminative features in a hierarchical way from lower to higher scales. This allows identifying the basic movement patterns (in the lower layers of the architecture) along with the combination of several movements (in the higher layers), thereby capturing multi-scale salient patterns characterizing the different activities across individuals. 

The work most related to ours is the CNN proposed by Yang et al. [19]. This evaluated the hand gesture recognition problem [23] using recordings from body-worn sensors and on the opportunity activity recognition dataset [24,25] that includes activities in the kitchen, monitored by a variety of body-worn, object-based, and ambient sensors. The authors in [26] experimented with the use of deep CNNs in human activity recognition using data from smartphone sensors. They report increasingly better performance with the addition of new layers, while complexity is successfully decreased, ending up as an effective solution for smartphone-related activity recognition systems. In another work [20], a shallow CNN was used with a weight-sharing technique on accelerometer signals. This showed improved classification over previous techniques, such as PCA based on empirical cumulative distribution estimation and k-nearest neighbor classification. Evaluation was based on recordings collected using a cell phone in a controlled laboratory environment [27]. Moreover Jiang and Yin [22], aiming to exploit 2D CNNs, stacked the raw signals row-by-row such that every signal sequence becomes adjacent to every other sequence creating a 2D image. Then they calculated the magnitude of the 2D discrete Fourier transform of this 2D image and used it as input to a deep 2D CNN. A deep belief network (DBN) [28] was investigated for ADL classification in [29], however, these networks are fully connected and therefore do not capture local dependencies of the signals. A different approach was reported in [30], where a transfer learning methodology was proposed for activity recognition without using new labeled data. This approach is based on measuring the correlation between observations acquired from an old sensor for which trained models are available, and observations from a newer sensor. This multi-view learning technique achieved high accuracy and is applicable in both batch and online modes.

Furthermore, Ordóñez and Roggen [21] used convolutional and long short-term memory (LSTM) recurrent units to model the temporal dependencies for multimodal activity recognition showing improved performance over baseline CNNs. An interesting approach in handling the heterogeneity of data in the context of human activity recognition is reported in [31], where a combination of convolutional and long short-term memory layers forming a deep network serve as a fine-tuning process to efficiently learn new settings of an existing system. More details on the effectiveness of different network architectures can be found in [32], where deep, convolutional, and recurrent approaches are compared using recordings of physiological activity captured by wearable sensors.

### 1.2. Challenges in Activity Recognition Using Wearable Sensors

Moving from a laboratory environment to real-life experiments, researchers have to deal with numerous obstacles that they must overcome, concerning mostly the devices used to monitor older people. A first challenge appears in case the device is not placed with standard orientation, causing rotation of axes. For sensors such as the accelerometer, orientation plays a significant role in recognizing the subject’s activity. Thus, a misplacement of a wearable device could easily disorientate the data analysis. It should be noted here that research has been conducted in the past regarding the sensor displacement problem, e.g., in [33] the authors present a benchmark dataset to examine the performance of activity recognition algorithms. Nonetheless, both the problem of inconsistent sensor placement and the lack of uncontrolled environment data still exist, thus need to be addressed.

Another issue occurs when a different type of sensor is used across individuals (e.g., accelerometers with different technical characteristics), or the sensors are placed in different locations on the body. This is possible when data from different clinical centers are combined or for example, after updating hardware and software components, since it causes lack of uniformity in the dataset adding barriers on inference and modeling. When activity annotations are used for model construction (e.g., in supervised learning settings), additional challenges come from the inter- and intra-rater variability, the rater’s subjectivity and the interactive nature of the annotation process, especially when frail individuals are instructed and monitored. A relevant work that aimed at integrating new sensors to an existing activity recognition system is reported in [34], where a semi-supervised graph method was used to incorporate the information provided by the new sensor and improve the system’s performance. The list of challenges could be really long, but we focus on the aforementioned issues since they are likely to occur in uncontrolled sensor systems, such as the one used in this work. Retrospective solutions on how to address these issues during data analysis are proposed and discussed in the subsequent section. 

### 1.3. Aim of Current Study

The current study builds upon and extends our previous work [8,12]. The workflow includes conventional steps, such as noise reduction, feature extraction, data imputation to fill in missing values, data standardization and classification with or without embedded dimensionality reduction. The main contribution of this work involves the integration of all methodological steps into an end-to-end platform performing activity recognition for older individuals based on only a small number of annotated examples and under challenging conditions, mainly due to data variations and erroneous measurements. Additionally, this end-to-end system is compared with three advanced CNN architectures, each of them based on different underlying assumptions (such as existence of correlation across sensors or correlation across axes within the same sensor). In more detail, the individual contributions of this paper can be summarized as follows:

- A SVM-based classification method is developed and assessed exclusively on older people’s recordings from a wearable sensor in everyday life conditions.
- Variations of the basic model are proposed to address device-relative issues that are due to data acquisition based on two different wearable devices, as well as their possible misplacement, during monitoring of physiological activity.
- The subject-specific prediction models of our previous work [12] are replaced with subject-independent models to avoid the laborious pre-training phase for every new-coming subject.
- Temporal consistency criteria are enforced to improve the predictions’ robustness.
- Three different convolutional neural network architectures are developed and applied to the same ADL recognition problem improving classification accuracy over our standard approach [8].
- Advanced Bayesian optimization is exploited for efficient hyper-parameter tuning.

The remainder of this article is structured as follows. In Section 2, details on both methodological approaches are provided followed by the experimental procedure and data description. Implementation details for each approach (based on SVM and CNNs) are provided along with the corresponding results in Section 3. Discussion on the results of the current study and related work are presented in Section 4, while some final conclusions are summarized in Section 5. 

## 2. Materials and Methods

The main device used to record the physiological signals of older people is a wearable solution that takes its origin from a previously developed product of Smartex (Pisa, Italy) [35], with a further integration of some inertial measurement units (IMUs) in order to have information of higher quality with regard to movement analysis. Together with data on movement, posture and physical activity, it also monitors the cardiac rhythm and respiration. The data collection was part of a European research project, FrailSafe [9]. For the current study only movement, and posture information was used; specifically, the standard approach was based only on 3-axial accelerometer recordings, whereas for the CNNs we exploited also the 3-axial gyroscope and magnetometer recordings, thus 9 channels in total. This choice was made on the one hand due to the inherent weakness of standard classifiers in handling high dimensional feature vectors, and on the other hand due to computation and performance-related constraints imposed on the standard approach which was also part of the online analysis module of the FrailSafe platform.

The purpose of the classification procedure was to discriminate basic movement activities, such as sit/stand, laying, walking, walking upstairs/downstairs, and transitional state, and store them in the user profile [36], in order to tailor the treatment or recommendations according to the older person’s physical condition. The activity recognition scheme includes standard modules of preprocessing, feature extraction and classification of new instances using previously trained models, along with dedicated processing steps that deal with data inconsistencies. In the next section the individual steps of the augmented standard approach will be described, whereas in Section 2.2 the three investigated deep learning schemes will be presented along with the applied transfer learning and data augmentation techniques. 

### 2.1. The Augmented Standard Approach

**Figure 1:**
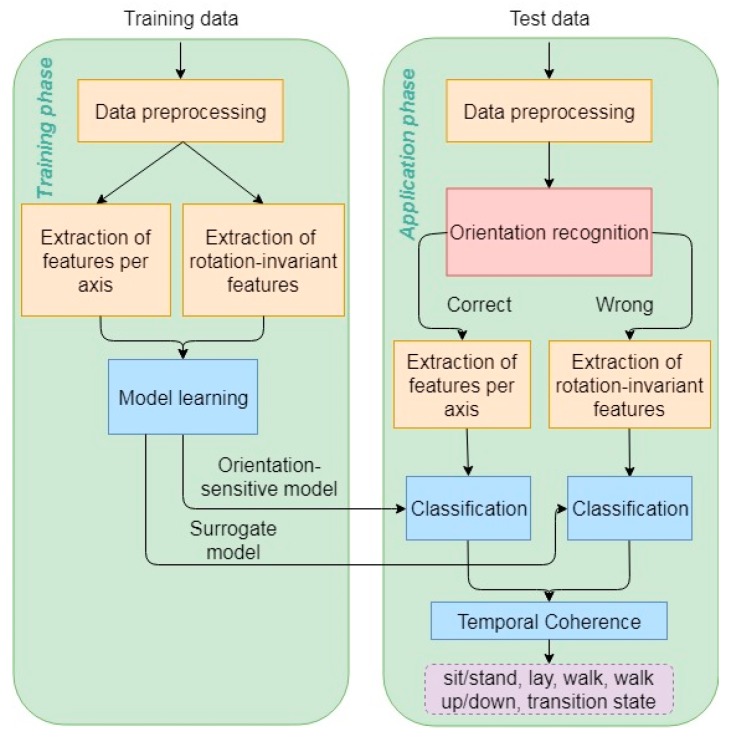
Pipeline of activity recognition methodology.

The classification scheme is based on previously reported work [12] but is augmented to account for data inconsistencies [8], while additional post-processing enforces temporal consistency in the activity predictions. A schematic representation of the complete methodology is illustrated in Figure 1. Explanations and reasoning on the individual methodological components are provided in the following paragraphs. 

#### 2.1.1. Preprocessing and Feature Extraction

The preprocessing procedure involves the separation of body acceleration from gravity acceleration, as reported in [37]. Specifically, the raw 3-axial signals from the accelerometer were initially preprocessed using low-pass filtering to separate the body and gravity acceleration components. The accelerometer Jerk signals were also calculated, as well as the magnitude of the tri-axial signals. The recordings were then split into fixed-width sliding time windows and a set of statistical features were calculated for each time window. These were a subset of the proposed features reported in [37], namely: mean value, standard deviation, median absolute deviation, largest value in array, smallest value in array, signal magnitude area, energy measure, interquartile range, signal entropy, autoregression coefficients, correlation coefficient between two signals, index of the frequency component with largest magnitude, weighted average of the frequency components to obtain a mean frequency, skewness of the frequency domain signal, kurtosis of the frequency domain signal, and energy of a frequency interval within the 64 bins of the fast Fourier transform (FFT) of each window. The resulting feature vector contained 254 features. 

Subsequently, the features were normalized, in order to avoid skewing the analysis by specific features’ scale. This was achieved by applying the widely used z-score normalization (standardization), that centers the features at zero and scales them to have unit variance. The parameters that used to scale the training features were stored in a data structure to be subsequently used for the standardization of the test features.

#### 2.1.2. Reducing Differences across Devices

As discussed previously, performing experiments with sensors of slightly different technology, or with a different sensor placement prohibits the use of a uniform classification model. In particular for the data used in this study, we explored whether any differences occurred between recordings from the two incorporated devices—a wearable wellness system (WWS) and a wearable WBan system (WWBS)—by asking two young volunteers to perform daily activities while simultaneously wearing the two devices used in the study placed at the center and laterally of the chest, respectively. It was revealed that a scaling difference occurred between the two types of measurements. To address this issue, we define a reference space and normalize all recordings with regards to the reference measurements using a baseline correction technique. The baseline was defined as the mean value of time segments with small standard deviation for each axis. Correction was performed by aligning the baseline of each new recording to the reference baseline of the corresponding channel. After baseline correction, the classification model to be used is selected according to the rules identifying errors in sensor’s orientation, as described in the next section.

#### 2.1.3. Resolving the Rotation of Axes Issue

To overcome the challenge of axes rotation, we investigated the possibility to automatically identify mis-oriented device’s data and map them back to a reference space. Although some heuristic rules helped us to recognize mis-orientations in most cases, no robust automatic technique was found to be always successful. Thus, we decided to extract also rotation-invariant features and build a substitute classification model. To achieve this, each triplet of features extracted from the three axes, X, Y and Z, was reduced to only one feature corresponding to the magnitude of the three-dimensional vector. These features are insensitive to the orientation, and thus to the misplacement of sensors.

Although the rotation-invariant features are preferable, in the case of inconsistent data they are expected to have lower predictive value, because some activities, such as walking, are strongly related to a particular axis. Therefore, it would not be the best practice to replace the detailed model by a less complex one overall. Accordingly, we had to deal with the problem of automatically recognizing whether the wearable device had been misplaced or not, and accordingly select the most appropriate model. The problem was addressed by learning the distribution of measurements in the reference space (correct orientation) defined by the training set. A two-step heuristic approach was introduced for that purpose. The first rule determines whether the vertical axis (axis X), which is the most prominent axis due to gravity, coincides with the one used during training. This was achieved by outlier detection assuming that the largest amount of measurements (80th percentile) of test and reference data should be in the same range. If so, then the axis-dependent model is selected for classification, otherwise the second rule is examined. 

The 2nd rule is used to examine more thoroughly cases in which the signal has a shifted baseline value, and therefore might fall outside the predefined range. The signature for the recording is based in this case on the overall distribution of measurements along all three axes and specifically on the 3D histogram of raw values (within ~1-h interval). Outlier detection is performed by computing the pairwise distance between the test signal and each of the reference signals (from the training subjects). If the minimum distance is smaller than a threshold (*θ*), the axis-dependent model is selected, otherwise classification is based on the axis-invariant scheme. The threshold *θ* was determined by examining the normal variation, i.e., it was equal to the maximum within-training-subjects’ distance. The chosen distance metric was a 3-D version of the Kolmogorov–Smirnov distance [38]. 

#### 2.1.4. Classification and Feature Selection 

The normalized feature vectors of the training samples were used to train a classification model that can be later used to classify new instances. A SVM classifier with radial basis function (*rbf*) kernel from the LIBSVM library [39] was used to train the orientation-sensitive as well as the rotation-invariant classification models. SVMs find the hyperplane that separates the classes by maximizing the margin in between. They have been proved very successful in pattern classification from physiological signals [40,41,42]. Grid search on parameters *C* and *gamma* of the *rbf* kernel was performed, in order to achieve optimum cross-validation accuracy on the training set. To deal with imbalanced classes (since the number of samples that corresponded to each class was different), we used weights, inverse proportional to the class size, in the classification function.

Furthermore, we performed dimensionality reduction and removal of irrelevant features using the Relief-F algorithm [43], due to its good performance in multiclass problems [44]. 

#### 2.1.5. Enforcing Temporal Coherency of Activities 

In order for the classification results to be physically plausible, we made the assumption that the minimum duration of an activity should not be less than a threshold. This assumption was made to ensure that the predictions describe coherent transitions between activities. To that end, we “smoothed” the activity signal using a moving majority voting filter of size equal to the predefined minimum duration of each activity. The idea is similar to the smoothing algorithm in [45] which aimed to remove potential wrong predictions in the form of impulse noise, resembling low-pass filtering. We used a value of 4 sec, which was considered as appropriate for not very sudden activity transitions, as is the case for the movement of older people, and since abrupt actions, such as unexpected falls, are not part of the analysis. 

### 2.2. Deep Learning Approach

In addition to the previous classification framework, we studied the performance of convolutional neural network architectures in recognizing movement patterns of older people. The aim was not to substitute the standard classification framework, but rather to better understand the potential of deep learning techniques. In fact, the former classifiers coupled with ranking-based feature selection can be integrated much easier into artificial intelligence modules of monitoring systems (such as that of the FrailSafe project) than deep networks, due to their limited dependencies, small computational cost and reduced requirements of large numbers of annotated data for training. However, deeper architectures offer prospective of further exploitation when databases get richer and also provide advantages of seamless analysis avoiding the tedious step of dedicated feature extraction, which if proven successful, accelerates the deployment phase.

We investigated three convolutional neural networks that are optimized and applied for discriminating the basic movement patterns of older people. The CNNs take as input overlapping time windows of the multi-channel recordings. Depending on the dimensionality of the convolutional filter and the multi-dimensional signal representation, various architectures are defined that process the multi-channel information in a different way, thereby affecting the networks’ performance. However, the common aspect in all three architectures is the extraction of translation-invariant patterns by applying the processing units in CNN along the temporal dimension and by sharing the units among multiple sensors [19]. The time window used to extract the samples for classification should be large enough to contain sufficient information for the discrimination of the activity, but not too large in order (i) to avoid including more than one activity within its time span and (ii) not to increase the latency (buffering time) of real-time recognition in data streams. The amount of overlap between the extracted time windows also affects the performance. On the one hand high overlap allows us to capture the smooth transition between activities and also ensures that each activity pattern is included within some time window (and not divided across time windows), but on the other hand large overlaps increase significantly the computational cost while the handling of very similar samples does not provide a substantial benefit. We used for all experiments a time window of 68 time points corresponding to 2.72 sec with 50% overlap, in accordance to previous work [12]. Since more than one activity classes might be present in the sliding window technique, the sample’s annotation during training follows the majority rule. 

The three deep network architectures are relatively shallow since the available training samples were not enough to train deep architectures efficiently without jeopardizing their generalization ability. The layers of a CNN have neurons arranged in three dimensions, that are denoted as *width*, *height* and *depth* (W×H×D). Note that the word *depth* here refers to the 3rd dimension of an activation volume, not to the total number of layers in a deep network. The main difference of the networks is in the first (input) layer, which affects the number of computed feature maps, as detailed below.

- **CNN1**: 1D convolution is performed on the input data along the temporal dimension only, with a convolutional kernel of size 5×1. The data consist of 9 channels, which are the recordings of the 3 tri-axial sensors, arranged in the depth dimension (D=9).
- **CNN2**: 2D convolution is performed with a 5×3 convolutional kernel along the temporal dimension and the sensing modality by stacking the different sensors (accelerometer, gyroscope, magnetometer) row-by-row and arranging the x, y and z axes in the depth dimension (D=3). Since the height of the input data equals the height of the convolutional kernel (H=3), the 2D kernel slides only along the temporal dimension.
- **CNN3**: Following the idea of Jiang and Yin [20], we created a “2D signal image” by stacking the input channels row-by-row with repetition, such that every sensor becomes adjacent to every other sensor. Specifically, we arranged the recordings of the accelerometer in x, y, z, the gyroscope in x, y, z and the magnetometer in x, y, z, and introduced again the accelerometer in x, y, z, thereby creating a 2D signal image of height H=12. By using a convolutional kernel 5×6, all different sensor combinations were possible (accelerometer with gyroscope, gyroscope with magnetometer and magnetometer with accelerometer). The convolutional kernel this time slides along both axes (over time and over sensors). By using a stride of 1×3 the bundles of x, y, and z channels were kept together. The depth dimension is vanished in this architecture (D=1).

**Figure 2:**
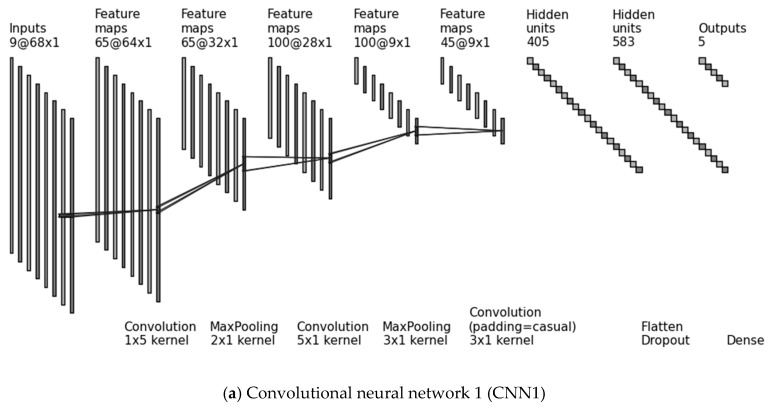

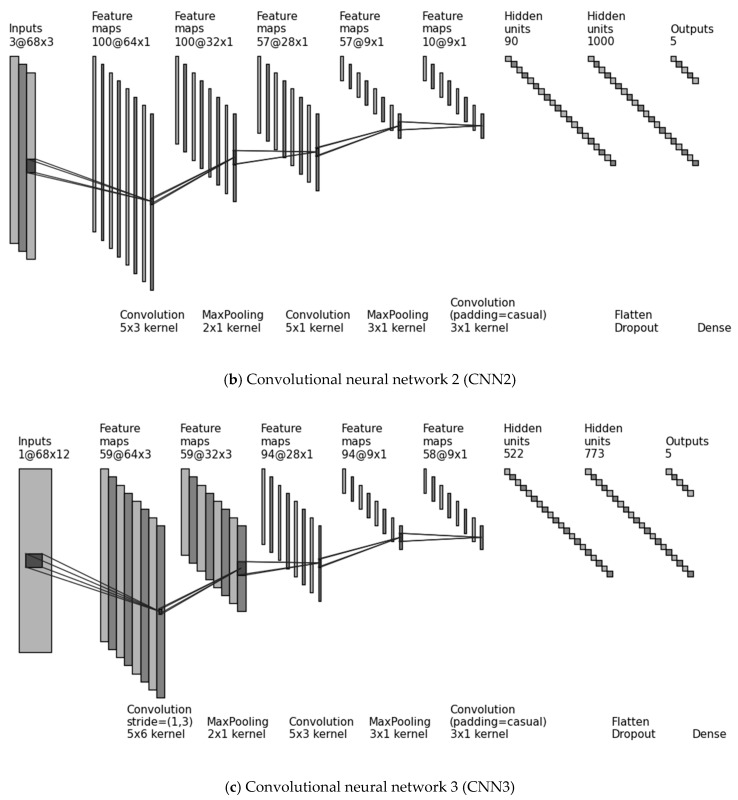
The 3 investigated optimized deep network architectures (CNN1, CNN2, CNN3). Each convolutional layer is followed by a normalization layer and a rectified linear unit (ReLU) activation unit, which are not illustrated in the figure due to space limitations. The numbers before the character “@” indicate the depth dimension, whereas following the character “@” the size of the feature maps (W×H).

The three CNNs are illustrated in Figure 2. The convolutional kernels are the same for the subsequent layers. Each convolutional layer is followed by a normalization layer and a rectified linear unit (ReLU) activation unit, which are not illustrated in Figure 2 due to space limitations.

#### Implementation Details

The exploration and choice of the networks hyperparameters was aided by the Bayesian hyperparameter optimization platform SigOpt [46]. Bayesian optimization is very well suited for multi-parametric problems with costly objective functions when first- or second-order derivatives are not available, and has therefore lately become very popular for tuning the hyperparameters of deep networks [47]. SigOpt is a standardized and scalable platform that is accompanied by an application programming interface (API) facilitating the generation of well performing models. It also allows parallelization for faster evaluation.

The CNNs were implemented in TensorFlow [48] using Keras [49] and CUDA. The experiments were executed on an Intel(R) Xeon(R) @ 3.70GHz processor with 8GB RAM and a GPU (Titan Xp 12GB VRAM (NVDIA, Santa Clara, CA, USA)). 

### 2.3. Experimental Procedure

The recordings were obtained from older people (age: 70–92 years) who participated in the FrailSafe project [9]. All participants were instructed to perform a set of activities while wearing one of the two devices (WWS or WWBS). The IMUs included a 3D accelerometer, 3D gyroscope and 3D magnetometer with a sampling rate at 25 Hz. The protocol was performed in clinical centers of three different countries, and included the following actions:

- Standing for 1 min;
- Sitting for 1 min;
- Walking for 1 min;
- Walking upstairs for 30 s;
- Walking downstairs for 30 s;
- Laying for 30 s.

**Figure 3:**
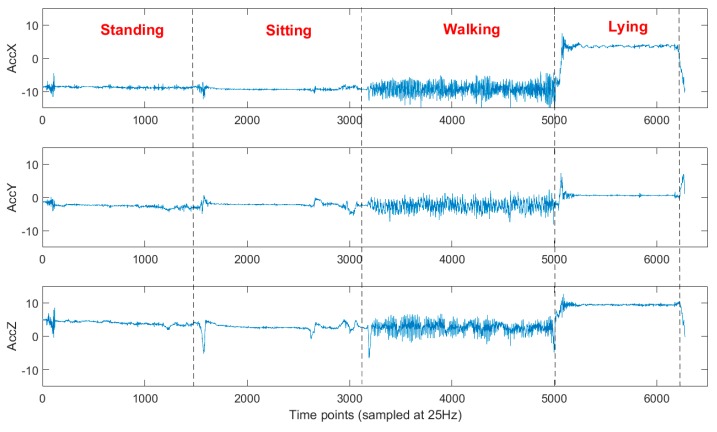
Acceleration signals while performing activities of daily living (ADLs).

All participants performed all activities except of upstairs and downstairs walking which was only performed if stairs were accessible by the subjects in their residence. Times were recorded by the medical instructors while the participants performed the ADL protocol. A prediction model was trained using part of the annotated data and assessed on the left-out data. Four-fold cross-validation on the training set was used to optimize the parameters for both classification models. The recordings of the subjects selected for training had the same orientation, which was used as reference space (with -X denoting the vertical axis). An example of the raw acceleration signals during different activities is illustrated in Figure 3.

Although six activity classes were initially defined, the classes *sitting* and *standing* were merged into one class, as well as *walking upstairs* and *walking downstairs*. This was performed based on previously reported work that suggests that these classes are not easily separable [12]. Additionally, the time windows corresponding to the first five seconds of the beginning of each activity, were automatically annotated as “transition state”, to indicate the time required to switch between two different activities. To that end, the final classes were: *sitting/standing, laying, walking, walking upstairs/downstairs, transition state.*

## 3. Results

### 3.1. Augmented Standard Approach

The classification framework was evaluated on accelerometer signals of 20 older individuals (17 females and 3 males), categorized according to Fried’s criteria [50] in 10 non-frail and 10 pre-frail subjects. The annotations of 8 subjects, all from the same clinical center, were used to train the classifiers, while the rest of the subjects were left out for testing. The selection of training subjects was based purely on the consistency and quality of recordings criteria. The idea was to construct a coherent subset of annotations and to use it as a reference space on which any incoming data would be mapped. Details on the data in respect to incorporated devices, orientation of sensor according to visual judgment (ground truth is unknown), and data split are shown in Table 1.

A grid search with internal four-fold cross-validation was performed on the training set to optimize the SVM hyper-parameters (C and gamma), as well as the most important variables based on feature selection. For the standard model, the best accuracy was achieved for C=0.01 and gamma=3.12 using only the 10 (out of the initial 254) higher-ranked features. In the case of the rotation-invariant model, a total of 40 out of initially 90 features were selected using the same feature selection technique and C=0.1 and gamma=2.24. We observed that features from the Y-axis did not contribute to the final classification model. 

Using the selected features and the optimized hyper-parameters, the cross-validation accuracy of the standard classification approach, computed as the average (across folds) percentage of correctly classified validation instances, was 89.46%. The rotation-invariant model on the same subjects obtained a smaller cross-validation accuracy (75.56%), as expected. Since the recordings used for training did not show any visible rotation errors, the removal of orientation cues, critical in the case of sensor inconsistencies, led to information loss and, therefore, to less-sensitive solutions. Based on this observation, the necessity of a rule to automatically identify the orientation and accordingly select the most appropriate model, became evident.

Furthermore, in order to obtain a final prediction model, we used all 8 training subjects to retrain the SVM classifier using the previously selected features. Τhe overall accuracy of the end-to-end pipeline was assessed on the 12 test subjects (not used during training) coming from different countries and whose recordings were acquired by wearing any one of the two devices, with the sensors placed correctly or rotated, and by letting the algorithm decide which model to use. The average accuracy was 71.8%. The accuracy drop might be either due to failure to recognize the orientation of sensors and thereby to select the most suitable model, or it might be due to limitations of the classification mechanism itself.

In the following, in order to better appreciate the effect of the different steps in the pipeline, we first selected 8 (out of the 12) test subjects wearing the latest of the sensor devices (WWBS) and whose recordings were acquired by placing the device according to the reference orientation. These subjects were from different clinical centers than the ones used in the training set. The classification accuracy of this independent test set reached 81.7%. It is evident that the effect of orientation errors is quite significant since it causes an accuracy drop of ~10%. The mean confusion matrix is illustrated in Table 2.

It appears that sit/stand is the most easily predictable activity by the model, since it is correctly classified in almost all cases. On the opposite the activity of walking on stairs (up/down) was completely misclassified as walking forward/backward, which might be explained by the very small size of walking up/down samples (one subject was available for training and one for testing). It might also indicate high similarity of walking patterns of the elderly, on the floor or in stairs, both characterized by slow speed and possibly instability. Class walking has some false negatives identified mostly as transition between activities, which is not surprising since a transition state is not a class with a standard pattern. Similarly, the class transition is intermixed with all other classes. This might be also attributed to imperfect annotations of the transition samples, extracted within constant time windows (located at the beginning of each activity) without any visual inspection or individualized correction. 

Furthermore, in order to assess the effect of using an alternative, rotation-invariant model, we used the recordings of the 2 test subjects using the WWBS device with orientation errors during acquisition, and also manually induced rotation in several ways on 6 other test subjects that were also using the WWBS. The mean classification accuracy for these 8 subjects was 70.31%. To better interpret the rotation-invariant models’ performance, we compare the classification accuracy of these inconsistent recordings when applying the orientation sensitive model and the current surrogate model. The results are depicted in Table 3. It is evident that the surrogate model boosts the accuracy significantly, thus being effective in addressing the rotation of axes issue. 

Finally, regarding the evaluation of the baseline correction technique for data acquired by different devices, we used recordings acquired with the WWS device from 4 participants (2 elderly from the test set and 2 additional young volunteers) and assessed the classification. The mean accuracy was initially 37.5% with the orientation-sensitive model (trained with data from the newer device), while after performing baseline correction it was increased to 61.7%. The results highlight the importance of baseline correction as data standardization step.

### 3.2. Deep-Learning Approach

**Figure 4:**
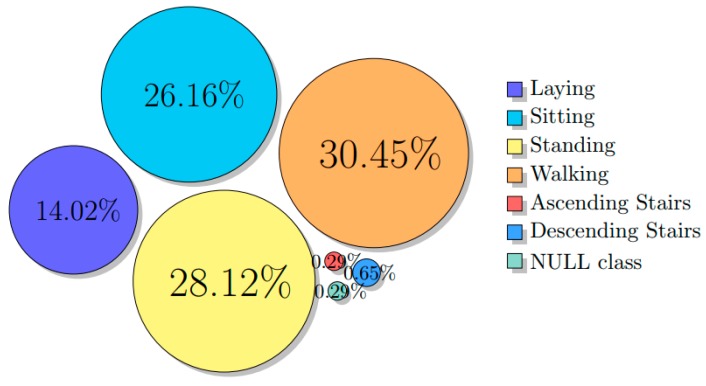
Samples distribution across classes.

The deep learning approach aimed to examine an alternative way of ADL classification that circumvents the need for dedicated feature extraction. Since the scope here was not to test the requirement of appropriate data manipulation techniques handling inconsistencies, we excluded data from the outdated device (WWS) and manually rotated the axes of the sensors whenever a misplacement was identified by visual inspection. Moreover, we used more data that were not available by the time of the development of the standard approach constructing a dataset of 23 subjects in total. We used 16 of them for training, in order to approximate the deep learning requirements of large number of annotations, and 7 subjects for testing. The recordings in both groups (training and testing) included recordings from different clinical centers in order to capture the data variability. The distribution of the different class labels is illustrated in Figure 4 where the NULL class represents the transitional class.

**Figure 5:**
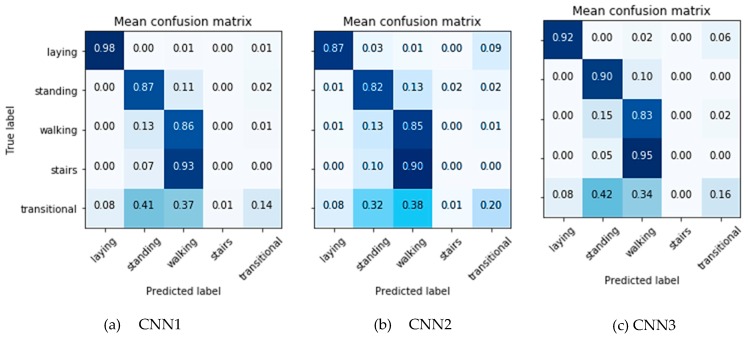
Average confusion matrices of classification of the test set using the 3 investigated deep network architectures.

The hyper-parameters were tuned by performing 4-fold stratified cross-validation on the training set using Bayesian optimization. The obtained values and their contribution in the classification performance are shown in Table 4. For each fold, the optimized CNN was then assessed on the independent test set. The average (across the folds) classification accuracy for training and test sets is depicted in Table 5, while the average confusion matrices for the 3 networks are shown in Figure 5.

## 4. Discussion

A direct comparison with other studies is not feasible due to differences in the experimental setup including the type of activity, as well as the use of different classification performance metrics. Nevertheless, the different approaches reported in this paper are compared in Table 6, in respect to incorporated sensors, classification technique, and performance. 

Other works report higher accuracy than the current work, but there can be many reasons for this. First of all, most studies use data from younger participants, selected to be in good physical health. This results to a more homogeneous group and allows the collection of a larger number of samples. Since our work is targeted to the ageing population, functional status and age variability are confounding factors when building a monitoring system for older people. Second, we use a sensor in a single location (sternum), whereas the combination of more sensors could affect the classification performance, especially if located in different parts of the body. Third, the data of this study were acquired in each participant’s house using a prototype research platform (FrailSafe system) based on different versions of sensors and with possible measurement errors, whereas most of the studies report accuracies in controlled, simulated or laboratory settings with consistent data. Appropriate processing steps, such as orientation recognition and classifier adaptation, were introduced to mitigate the effects of misplacement, mis-orientation and device-related variations in the data, reducing the measurement bias and automating the analysis within this end-to-end platform. 

Regarding the problem of increased data variation which is present in our dataset, multiple techniques have been proposed in the literature to address this issue. More specifically, transfer-learning methods can be used in order to reduce the requirement of a large number of labeled data, as reported in [51]. The authors propose a transfer learning-oriented methodology to build personalized affective models without labeled target data. The latter is accomplished either by exploiting a shared structure underlying source subjects and target subjects, or by training multiple classifiers on source subjects and transferring the classification parameters to the target subjects. A different approach to address the same problem includes the use of semi-supervised techniques. In a relevant study [52], a semi-supervised clustering methodology is proposed for physical activity recognition. The approach is able to capture potential shifts in the subject’s behavior, e.g., falls, with overall accuracy, while requiring a small number of labeled data. Such techniques are valuable, nevertheless in our case the data variations mostly come from device-related differences, rather than subject-to-subject variations.

Finally and more importantly, our methods were assessed by cross-validation on the subjects, i.e., the model was trained using measurements from subjects not used during testing. This gives us an estimate on the method’s accuracy when applied on recordings of new subjects. By contrast, other methods either do not report cross-validation results [11,16,17], or split the data segments into training and testing without considering whether segments from the same subject are included in both training and test sets [15]. This can increase the accuracy significantly since neighboring segments in the same recording can have very similar patterns. Similarly, in our deep-learning approach, the training accuracy can reach the value of 97.9% for CNN2 when transfer learning is performed, but we do not consider this value as indicative of accuracy since it does not generalize to new coming data. A relevant type of analysis involves the construction of subject-specific models [12], where a unique model is created for each subject using part of the data for training and the remainder for testing. The accuracies obtained are expected to be higher and not directly comparable to those of subject-independent models, as the models developed in this work. 

## 5. Conclusions

In this paper we presented a system recognizing basic physical activities from wearable sensors, with respect to challenges arising from device-generated or human-related parameters. Data from older participants with different levels of frailty and functional conditions were used to train and assess the end-to-end modeling framework developed to address those challenges. Classification was performed by standard machine learning, as well as deep learning techniques, demonstrating a slight advantage of the latter. Overall promising results support the use of the proposed activity recognition scheme for unobtrusive monitoring of older people. Future work includes the investigation of data augmentation and transfer learning techniques to allow the exploitation of available databases from younger people, and thereby to improve the performance of the deep networks.

## Figures and Tables

**Table 1:** Characteristics of recordings and data split for evaluation. Orientation consistency could not be visually determined across devices, and is therefore not marked.

ID	WWBS	Probably Correct Orientation	Used for Training	Older Adults
3087	yes	yes	yes	yes
3098	yes	yes	yes	yes
3104	yes	yes	yes	yes
3116	yes	yes	yes	yes
3117	yes	yes	yes	yes
3593	yes	yes	yes	yes
3600	yes	yes	yes	yes
3601	yes	yes	yes	yes
1117	yes	yes	no	yes
2101	yes	yes	no	yes
2113	yes	yes	no	yes
2615	yes	yes	no	yes
3084	yes	yes	no	yes
3091	yes	yes	no	yes
3112	yes	yes	no	yes
3118	yes	yes	no	yes
1507	yes	no	no	yes
1538	yes	no	no	yes
2094	no	—	no	yes
2102	no	—	no	yes
9000	no	—	no	no
9001	no	—	no	no

**WWBS:** wearable wireless body area network system.

**Table 2:** Mean confusion matrix on the test set using the latest sensor device.

**Actual**	**Predicted**					
	Classes	Sit/Stand	Laying	Walking	Walking up/down	Transition
	Sit/Stand	96.08	0	0.76	0	3.16
	Laying	0	86.75	1.65	0	11.60
	Walking	8.26	0	74.33	1.56	15.85
	Walking up/down	0	0	100	0	0
	Transition	36.07	2.73	18.03	0	43.17

**Table 3:** Performance of the orientation-sensitive model and rotation-invariant (surrogate) model in case of recordings with axes’ rotation.

Subject	Classification Accuracy %		Increased by %
	Orientation Sensitive Model	Surrogate Model	
1	19.4	60.2	40.8
2	19.6	60.4	40.8
3	23.1	72.6	49.5
4	29.8	78.0	48.2
5	25.3	64.5	39.3
6	7.0	69.2	62.1
7	7.2	87.1	79.9
8	25.4	78.5	53.2

**Table 4:** Optimized hyper-parameters using SigOpt (*SGD*: stochastic gradient descent).

	Hyper-Parameters	Values			Contribution in the Model		
		CNN1	CNN2	CNN3	CNN1	CNN2	CNN3
FrailSafe dataset	Batch	100	100	59	2.14%	1.50%	1.62%
	Dense Layer Size	583	1000	773	1.17%	1.82%	1.92%
	Dropout prob.	0.6	0.39	0.6	2.68%	2.20%	1.29%
	Epochs	100	100	100	3.90%	2.65%	3.76%
	Filter 1	65	100	59	2.38%	1.95%	2.06%
	Filter 2	100	57	94	2.12%	2.41%	1.88%
	Filter 3	45	10	58	1.78%	1.17%	1.29%
	Learning rate	0.0330	0.0480	0.1000	17.49%	9.46%	26.61%
	Regulariz. rate	0.0030	0.0001	0.0001	16.83%	9.32%	32.63%
	Optimizer	SGD	SGD	SGD	48.72%	67.52%	26.89%

**Table 5:** Average classification accuracy across in 4-fold stratified cross-validation.

	CNN1	CNN2	CNN3
Test Accuracy	81.91(±2.45)	78.49(±3.66)	82.47(±4.24)
Train Accuracy	90.64(±1.34)	90.86(±0.83)	91.84(±1.17)

**Table 6:** Works on physical activity recognition of older people in real-world settings.

Study	Sensor/Location	Measurement	Method	Cross-Val.	Inter-Subj.	Accuracy
Current study	IMU at sternum	acceler.	SVM	yes	yes	81.7%
		acceler., gyroscope, magnetometer	CNN3			82.47%
[15]	Smart watch	acceler., temperature, altitude	NNs, SVM	yes	no	90.23%
[16]	IMUs at sternum and thigh	orientation, acceler., angular velocity	Rule-based	no	no	97.2%
[17]	Instrumented shoes	foot loading, orientation, elevation	Decision Tree	no	no	97.41%

* Inter-subject analysis means the method is assessed on measurements from subjects not used during construction of the classification model. Cross-val.: cross-validation, Inter-subj.: inter-subject analysis*, IMU: inertia measurement unit
